# Structure of the acetophenone carboxylase core complex: prototype of a new class of ATP-dependent carboxylases/hydrolases

**DOI:** 10.1038/srep39674

**Published:** 2017-01-05

**Authors:** Sina Weidenweber, Karola Schühle, Ulrike Demmer, Eberhard Warkentin, Ulrich Ermler, Johann Heider

**Affiliations:** 1 Max-Planck-Institut für Biophysik, Max-von-Laue-Str. 3, 60438, Frankfurt am Main, Germany; 2 Laboratorium für Mikrobiologie, Fachbereich Biologie and SYNMIKRO, Philipps-Universität, 35032, Marburg, Germany

## Abstract

Degradation of the aromatic ketone acetophenone is initiated by its carboxylation to benzoylacetate catalyzed by acetophenone carboxylase (Apc) in a reaction dependent on the hydrolysis of two ATP to ADP and P_i_. Apc is a large protein complex which dissociates during purification into a heterooctameric Apc(αα′βγ)_2_ core complex of 482 kDa and Apcε of 34 kDa. In this report, we present the X-ray structure of the Apc(αα′βγ)_2_ core complex from *Aromatoleum aromaticum* at ca. 3 Å resolution which reveals a unique modular architecture and serves as model of a new enzyme family. Apcβ contains a novel domain fold composed of two β-sheets in a barrel-like arrangement running into a bundle of eight short polyproline (type II)-like helical segments. Apcα and Apcα′ possess ATP binding modules of the ASKHA superfamily integrated into their multidomain structures and presumably operate as ATP-dependent kinases for acetophenone and bicarbonate, respectively. Mechanistic aspects of the novel carboxylation reaction requiring massive structural rearrangements are discussed and criteria for specifically annotating the family members Apc, acetone carboxylase and hydantoinase are defined.

Aromatic hydrocarbons are one of the most abundant classes of organic compounds in nature. They are primarily produced by plants as soluble secondary metabolic products or as components of the structural polymer lignin^1^. Moreover, considerable amounts of toxic aromatic hydrocarbons, in particular, of the BTEX (benzene, toluol, ethylbenzene, xylene) group accumulate during industrial petroleum processing as ground water contaminants^2^,^3^. Their biodegradation is of formidable biological/environmental importance and is essentially performed by special microorganisms in aerobic and anaerobic habitats. For that purpose, nature developed various pathways and enzymatic machineries with novel catalytic capabilities to enable chemically challenging dearomatization, C-H or C-C cleavage reactions.

Ethylbenzene is aerobically or anaerobically catabolized via different pathways in various bacteria^4^,^5^. In most degradation pathways, the ethyl group of ethylbenzene is initially hydroxylated to 1-phenylethanol, either by a monooxygenase in aerobic bacteria^6^ or by the molybdenum enzyme ethylbenzene dehydrogenase in anaerobic bacteria^7^. The alcohol is subsequently oxidized further to acetophenone by an alcohol dehydrogenase^8^, and the ketone is carboxylated to benzoylacetate by acetophenone carboxylase (Apc) (Fig. 1a). Finally, benzoylacetate is activated to the CoA-thioester, which is then thiolytically cleaved to the products benzoyl-CoA and acetyl-CoA^5^,^9^. Apc is the key enzyme of acetophenone metabolism and has, so far, only been characterized from *Aromatoleum aromaticum* strain EbN1^10^, where it is specifically induced in ethylbenzene and acetophenone-grown cells. Apc consists of five subunits and dissociates into a highly stable, yet inactive Apcαα′βγ core complex and the Apcε subunit upon purification. The Apc core complex is organized as an Apc(αα′βγ)_2_ heterooctamer with a molecular mass of 482 kDa. It contains 2 Zn^2+^, although the activity of Apc is strongly inhibited by further added Zn^2+^ ions. Kinetic studies further indicated the requirement of the Apc core complex, Apcε, and ATP, but not of biotin, for catalytic activity. 2 ATP are hydrolyzed to 2 ADP and 2 P_i_ per one acetophenone carboxylated. In addition, uncoupled ATPase activity with either bicarbonate or acetophenone was detected in the absence of the second substrate^10^.

Apc of *A. aromaticum* is encoded by the genes *apcA-E* (coding for Apcα′ (70 kDa), Apcγ (15 kDa), Apcα (80 kDa), Apcβ (75 kDa) and Apcε (32 kDa), respectively) which are located in an apparent operon together with the gene for the following enzyme of the metabolic pathway, benzoylacetate-CoA ligase. Apcα and Apcα′ are structurally related and have a sequence identity of 30%. Sequence comparison studies revealed phylogenetic relationships between Apc, acetone carboxylases (Acx) and ATP-dependent hydantoinases/oxoprolinases (Hyd) with sequence identities between 20 and 30%^11^,^12^. However, the subunit compositions of the three enzyme types differ. Acetone carboxylases consist of three subunits and hydantoinases/oxoprolinases of only two. To work with a uniform terminology we define the two core subunits present in all enzymes of the apparent family as α and β, the short subunit present in Apc and Acx as γ and the additional subunits of Apc as α′ and ε, resulting in an αβ protomer for Hyd (from genes *hydA* and *hydB*), an αβγ protomer for Acx (from genes *acxA, acxB* and *acxC*) and an αα′βγε protomer for Apc (from genes *apcC, apcA, apcD, apcB, apcE*).

Acetone carboxylases (Acx) are organized as Acx(αβγ)_2_ heterohexamers. Upon carboxylation of acetone to acetoacetate by HCO_3_^−^, one ATP is either stepwise hydrolyzed to ADP and AMP plus 2 P_i_ by the enzymes from *Xanthobacter autotrophicus* or *Rhodobacter capsulatus*^13^,^14^,^15^, or 2 ATP are hydrolyzed to 2 AMP and 4 P_i_ without the transient formation of ADP by the enzyme from *A. aromaticum*^12^ (Fig. 1b). Hydantoinases/oxoprolinases occur as Hyd(αβ)_2_ heterotetramers that act on diverse substrates as cyclic amidohydrolases with concomitant hydrolysis of ATP to ADP and P_i_^16^,^17^ (Fig. 1c). These three types of enzymes, grouped to the hydantoinase/ketone carboxylase family, share the capability to activate a carbonyl group to a phosphoenol (or phosphoimidate) intermediate with ATP. In Apc and Acx the generated energy-rich phosphoenol intermediates are subsequently used to accomplish a new type of carboxylation and C-C bond forming reaction.

For a more profound understanding of the complex carboxylation reactions, detailed structural data are indispensable. In this report, we describe the first X-ray structure of a member of the hydantoinase/ketone carboxylase family, the 482 kDa Apc core complex, at ca. 3 Å resolution. The structural details allow to outline a first proposal of the catalytic reaction cycle of Apc. Carboxylation of inert methylketones is a basic chemical process which makes the Apc reaction also attractive for biotechnological applications.

## Results and Discussion

### Structure of the Apc core complex

The Apc core complex reveals an impressive novel protein structure with a size of ca. 250 Å × 100 Å × 100 Å (Fig. 2). It is present in the crystals as an Apc(αα′βγ)_2_ heterooctamer which confirms the previously reported oligomeric state derived from a gel filtration–based molecular mass determination in solution^10^. The heterooctamer is built up by a crystallographic two-fold axis from an asymmetric unit containing one Apcαα′βγ protomer. A relatively small protomer interface is formed exclusively between two Apcβ (1130 Å^2^; 4% of the surface area^18^). The Apcαα′βγ protomer is mainly held together by large contact areas between Apcα and Apcβ (3540 Å^2^). Apcα′ is located at the periphery of the Apc core complex and only attached to Apcα. The small Apcγ partly envelops Apcβ and is itself embraced by the extended C-terminal arm of Apcβ (Fig. 2). One Apcαα′βγ protomer is expected to contain one bound Zn^2+^ ion as detected by quantitative elemental analysis^10^. Although Zn^2+^ was not detected in the X-ray structure, two of the found mercury binding sites are also plausible for binding of Zn^2+^ ions. One mercury was found to be coordinated to Asp65, His123, Asp126 and His148 in Apcβ and a second to Cys22, Cys25, Cys75 and Cys78 in Apcγ (sFig. 1). We favour the latter as more probable physiological Zn^2+^-binding site in Apc because of the high conservation of the cysteines in Apc and Acx, its location in a classical Zn finger motif^19^ and the absence of Zn^2+^ in Hyd, which are lacking orthologs of Apcγ. As both potential Zn^2+^ binding sites are located far away from the proposed active site, the role of the Zn^2+^ ion is probably more structural and not directly related to the catalytic reaction.

The subunits of Apc are modularly built up of several domains arranged in a new topological manner. Based on the DALI^20^ and SCOP servers^21^ Apcα reveals a high structural relationship to Apcα′ reflected in a rmsd of 4.1 Å between the two subunits (96% of the Cα used) suggesting an evolution from a simpler common ancestor by gene duplication (Fig. 3). Their most prominent structural elements (Apcα: 1–90, 250–275; 275–510; Apcα′: 1–80, 215–465) belong to the ASKHA (acetate and sugar kinase/heat shock cognate/actin) superfamily^22^. Moreover, an α/β domain (Apcα: 91–249; Apcα′: 81–214) bridges the two domains of the ASHKA modules, and an α+β domain (Apcα: 511–630; Apcα′: 470–580) follows the ASHKA modules, respectively. In addition, the C-terminal β-barrel-like domains (Apcα: 640–715; Apcα′: 590–660) and the second domains of the ASHKA modules are linked via their central sheets (Fig. 3). Apcβ is composed of a Greek-key like β-domain (30–230), a mixed β-sheet domain (255–380), and a domain (385–605) with a novel fold (Fig. 4a,b). The novel domain can be subdivided in a distorted barrel composed of two β-sheets with a hydrophobic core in between and an unusual folding motif at the barrel bottom. Most strands of the two β-sheets converge to a bundle of eight glycine-rich segments (390–393, 422–427, 431–435, 507–510, 582–584, 475–479, 514–517 and 470–473) with polyproline II helical conformations (manuscript in preparation). In Apcγ, a β-hairpin, a β-meander and a four-strand antiparallel β-sheet are fused together. This subunit is tightly associated to Apcβ and does not assemble to a globular fold (Fig. 2).

### ATP and putative substrate binding sites of Apc

**Figure 1:**
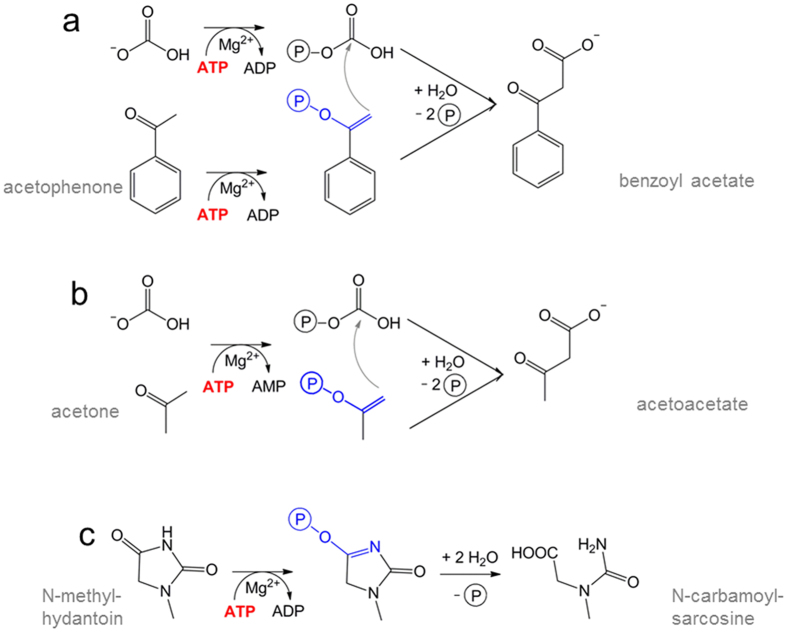
Reactions of Apc, Acx and methyl-hydantoinase. Apc requires the hydrolysis of 2 ATP to 2 ADP + 2 P_i_ (**a**), whereas most Acx hydrolyses one ATP to AMP + 2 P_i_ per carboxylase reaction (**b**). Hydantoinase hydrolyses ATP to ADP + P_i_ for imidate phosphorylation (**c**). In all three reactions the keto/enol equilibrium is shifted towards the enolate/imidate side by phosphorylation.

The X-ray structure of the Apc core complex revealed two ASKHA folding motifs as key components of Apcα and Apcα′ (Fig. 3) which could not be predicted on the basis of the primary structures. Superposition of the pantothenate kinase-ADP complex^23^ (pdb-code: 3BF1) or the actin-ATP complex^24^ (pdb-code: 4B1U) structures onto those of Apcα or Apcα′ basically indicated related ATP binding sites despite substantial deviations of the surrounding polypeptide architecture (Fig. 5). The conformations of the known signature motifs for ATP binding in ASHKA domains, ADENOSINE (GxxPGP), PHOSPHATE 1 (DxGGTxDDT) and PHOSPHATE 2 (DVGGT), are well conserved in Apcα and Apcα′, as well as in the modelled structures of Acxα and Hydα (see below). The cleft between the two ASKHA domains in the recorded structure of Apcα is open (Fig. 5a) and is expected to close upon ATP/ADP binding, as found in other structurally characterized ASKHA family members^25^. In contrast, the interdomain cleft in Apcα′ is closed. This is consistent with the presence of an apparently bound ADP, which has tentatively been fitted into residual electron density at the ATP binding site of Apcα′ (Fig. 5b). The structural data clearly suggest that both Apcα and Apcα′ are capable of ATP binding and thus presumably contain the active sites for the two ATP-dependent kinase reactions^10^. Previous kinetic studies on Apc demonstrated uncoupled ATP hydrolysis in the presence of ATP and either acetophenone or HCO_3_^−^ as sole substrates^10^. Independent phosphorylation reactions for each substrate are consistent with two separated ATP-dependent kinase sites as found in Apcα and Apcα′. Moreover, phosphorylations of carboxyl or hydroxyl groups are catalysed by the family members acetate or pantothenate kinase suggesting the suitability of the ASKHA modules for the chemically related reactions of Apc on ketones and bicarbonate. In analogy, the kinase reactions of Apc might proceed mechanistically by a nucleophilic attack of acetophenone in its isomeric form of an enol hydroxylate and of bicarbonate, respectively, onto the γ-phosphoryl groups of the respective ATP (+Mg^2+^) cosubstrates, forming phosphoenol-acetophenone and carboxyphosphate (Fig. 1a). This mechanistic scenario corresponds to the previously deduced pathway based on kinetic data and basic chemical principles^10^.

The known ATP binding sites in kinases also define the approximate substrate positions in the vicinity of the γ-phosphates of ATP^26^. In Apcα, the potential substrate binding site resembles similarly-sized pockets in pantothenate or acetate kinases^23^,^27^ (Fig. 5a) and is lined up by rather hydrophobic residues such as Leuα81, Pheα281, Asnα282, Proα285, Ileα333, Glnα344 and Thrα345. The solvent-exposed pocket is located at the bottom of a wide hollow with a depth of 25–30 Å formed by Apcα, Apcβ and Apcγ (Fig. 6a). In Apcα′, the potential substrate binding site is localized in front of the β-phosphate of the bound ADP. Compared to Apcα, the predicted binding site is also solvent-exposed, coated by a higher portion of polar residues as Gluα′248, Aspα′274 and Lysα′281 and narrower, perhaps partly due to the closed inter-domain cleft (Fig. 5b). Based on this qualitative analysis of geometric and polar properties, we tentatively assign the binding and activation of acetophenone to Apcα and that of HCO_3_^−^ to Apcα′. However, an unambiguous proposal would require structures of Apc–substrates/substrate analog complexes which are not yet established.

### Proposed carboxylation mechanism and a putative role for Apcε

Carboxylases are key enzymes in the biosphere because they catalyze the fixation of inorganic carbon (CO_2_) into organic matter and extend organic molecules by forming new carbon-carbon bonds. Nature developed diverse types of carboxylases. They can be subgrouped into reductive and non-reductive enzymes, which are further subdivided into carboxylases dependent or independent on biotin^28^. Apc and Acx belong to the non-reductive biotin-independent enzymes, which also include e.g. ribulose-1,5-bisphosphate carboxylase/oxygenase (RuBisCO^29^). Despite fundamental differences in overall architecture and active sites, these carboxylases share the basic strategy of their catalytic mechanisms consisting of the formation of an electron-rich enolate anion and an electrophilic carbon of CO_2_. Enolate intermediates are formed by ATP-independent and ATP-dependent tautomeric keto-enol shifts as catalyzed by RuBisCO^30^ and Apc^10^, respectively. The proposed ATP-dependent activation of HCO_3_^−^ in Apc is shared with biotin-dependent carboxylases. The generated carboxyphosphate might be channeled to the appropriate active site and decompose *in-situ* to the electrophilic CO_2_ which then reacts with the substrate either indirectly via the biotin cofactor^31^ or directly as in Apc^10^.

**Figure 2:**
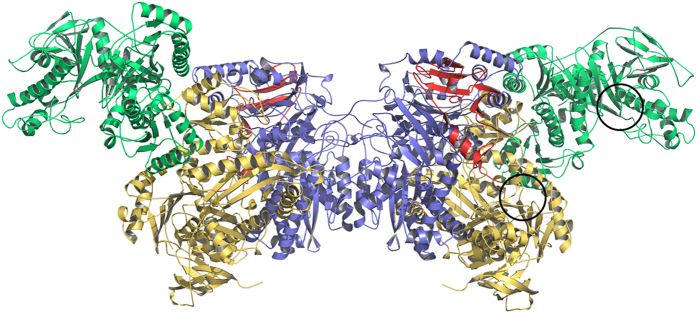
Structure of the Apc(αα′βγ)_2_ core structure. The interface subunit Apcβ is drawn in blue, the potential ATP binding subunits Apcα and Apcα′ in yellow and green and the small Apcγ in red. Apcα′ has no contact with Apcβ or Apcγ. The two potential ATP binding sites (black circles) are more than 50 Å apart from each other.

**Figure 3:**
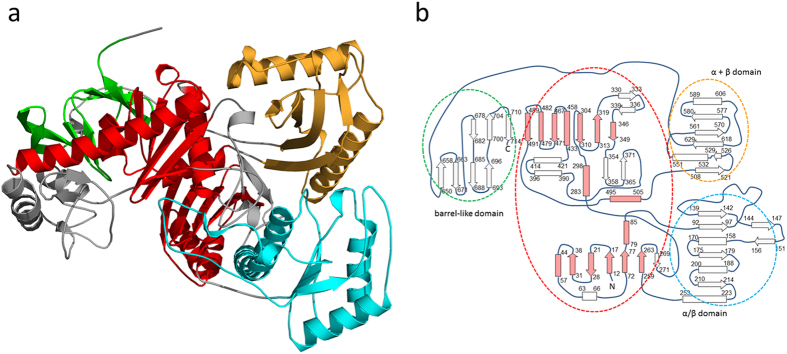
The Apcα fold. (**a**) Structure of Apcα. In Apcα and Apcα′ the ASKHA modules (in red) are the central components extended by several hairpin and helix insertions (grey) and flanked by three additional domains. The C-terminal barrel-like domain (green) associated with one domain of the ASKHA modules has no significant identity to any other protein of the pdb. The α/β domain (blue) and the α + β domain (orange) are structurally most related to the pyruvate dehydrogenase component E1 (pdb-code: 1W85) and a DNA polymerase IV domain (pdb-code: 4R8U), respectively. (**b**) Topology diagram of Apcα (strands as arrows, helices as boxes and polyproline II helices as rhombus) with the colours of the circles as in a. The canonical ASKHA module (in lightred) consists of two characteristic and closely associated domains forming an ATP binding site in between.

However, the apparent active sites for generating phosphoenol-acetophenone (Apcα) and carboxyphosphate (Apcα′) in the determined Apc core structure are ca. 50 Å apart from each other, which clearly precludes any direct interaction of CO_2_ formed from carboxyphosphate with phosphoenol-acetophenone (Fig. 2). Therefore, we expect a large-scale snapping-type displacement of Apcα′ relative to Apcα to occur during the reaction which forms a more closed conformation of the complex and positions the two activated substrates closer to each other. Such a rearrangement appears to be feasible due to the presence of a fixed interface consisting of the large contact area between the α/β domains of Apcα′(α′81–α′214) and Apcα, and the small contact area between the α/β domain and the exposed rest of Apcα′ (Fig. 3a). The loose fixation of the exposed part of Apcα′ to the residual Apc core complex (Fig. 2) is also reflected in its extraordinary flexibility (B = 145 Å^2^). The postulated movement of the exposed Apcα′ part into the wide hollow formed by Apcαβγ might be induced by the binding and activation of both substrates. However, due to steric hindrances the residual distance between the kinase sites of Apcα and Apcα′ in a modelled “tightened” complex is still predicted to be around 20 Å (from modelling results treating Apcα and Apcα′ as rigid-bodies). The apparent need of extensive conformational changes within the Apc complex during the catalytic cycle may implicate a role for the still elusive subunit Apcε. Apcε might be involved in the transitions between the “tightened” and “relaxed” forms of the Apc core complex and/or in the further bridging of the gap between the kinase sites. Consistent with kinetic and structural data we propose a first scenario for the mechanism of Apc (Fig. 6b): The reaction starts by binding acetophenone, HCO_3_^−^ and the respective ATP molecules which induce a conformational change of the exposed part of Apcα′ to move towards the wide hollow (Fig. 6b). Subsequently, Apcε binds to the Apc core complex in the closed “tightened” state and triggers the kinase reactions (Fig. 6b). As proposed for biotin-dependent carboxylases^32^,^33^, carboxyphosphate might be hydrolyzed on-site to the highly reactive CO_2_. For bridging the remaining gap between the active sites of Apcα and Apcα′, we speculate that the closed Apc complex with bound Apcε either forms a channel for carboxyphosphate or CO_2_ diffusion to its reaction partner (Fig. 6b), or induces additional conformational changes in the complex to provide a closer approach between the kinase sites of Apcα and Apcα′. Now, the electrophilic CO_2_ reacts with phosphoenol-acetophenone and the generated intermediate is hydrolytically dephosphorylated to the product benzoylacetate (Fig. 1)^10^. Finally, the Apc complex dissociates, releases Apcε, benzoylacetate, ADP and P_i_ (Fig. 6b), and leaves behind the “relaxed” open form of the Apc core complex (Fig. 6a). This postulated reaction cycle involves all five subunits of Apc, including Apcε which is apparently not attached to the core complex, but essential for the reaction. The required rearrangement of the core complex and the attachment/detachment cycle of Apcε may also explain the observed extreme tardiness of the reaction even with the purified and reconstituted enzyme^10^.

Compared to other carboxylases, Apc and Acx consume (at least; see below) two ATP equivalents per carboxylation event instead of one (as in biotin-dependent carboxylases) or none (as in RuBisCO). While activation of HCO_3_^−^ to carboxyphosphate is shared by other carboxylases, activation of the respective ketones to reactive enolate states with ATP appears to be essential because of the low acidity of the methyl protons (pK_a_ acetone : 19.2; acetophenone: 15.4) or methylene protons in case of longer substituents (pK_a_ butanone: 14.7; propiophenone: 17.6). We assume that this expensive activation strategy was specifically developed for carboxylating such chemically inert (alkyl)ketones. In other carboxylases substrate activation is achieved by biotin/ATP, by using electron-rich substrates such as phosphoenolpyruvate^34^, by metal ion ligation, and exceptionally by a carbamoylated lysine in RuBisCO^30^.

### The hydantoinase/ketone carboxylase family

Microbial genome analysis reveals the existence of a large new family of proteins containing highly related α and β subunits which are annotated as putative Hyd, Acx, and Apc complexes. *A. aromaticum* seems to be a special case of an organism that contains one member of all three branches of this enzyme family, namely Apc^10^, Acx^12^, and a γ-lactamase involved in anaerobic indoleactate metabolism^35^. Based on sequence comparisons and the structural data on Apc, we tried to extract features which are shared or distinct between the members of the three main branches of the enzyme family.

Firstly, we investigated the organization of the encoding genes, which appears to be conserved even if more than one enzyme of the family is encoded in the genome of a bacterial strain (sFig. 3). The gene clusters encoding hydantoinases/amidohydrolases consist of only two genes for the α (HydA) and β (HydB) subunits with a conserved gene order of *hydAB*. Gene clusters encoding acetone carboxylases exhibit the same order of the genes for α and β subunits (*acxAB*), but additionally contain a third gene for the γ subunit (*acx*C) downstream of *acx*B. Finally, gene clusters encoding acetophenone carboxylase or similar enzymes contain four or five genes in the order *apcABCD(E)*, which code for the subunits Apcα′, Apcγ, Apcα and Apcβ of the core complex, followed by the gene encoding the additional subunit Apcε. Note that the fifth subunit is not present in every predicted operon for an acetophenone carboxylase-like enzyme, and that the sequences of these subunits are much more divergent than any of the other subunits. Close orthologues to Apcε from *A. aromaticum* are only found in the gene clusters of *Nevskia ramosa* (43% identity) and *Rubrobacter xylanophilus* (26% identity).

Secondly, we constructed phylogenetic trees for the hydantoinase/ketone carboxylase family based on multiple sequence alignments^36^, which revealed a distinct clustering of the three functionally different subfamilies into three branches (sFig. 1). Moreover, several subclusters were identified in the respective branches, allowing to predict the function of so far uncharacterized enzymes. For instance, a number of predicted 2-oxoindoleacetyl-CoA γ-lactamases involved in indoleacetate metabolism^35^ form a well-defined cluster within the branch of Hyd variants, and the characterised Acx variants hydrolysing either one or two ATP per acetone to AMP and P_i_ appear to form separate subbranches^12^,^13^,^14^,^15^ (sFig. 1).

Thirdly, an approximate distinction between Apc, Acx and Hyd is possible by the different lengths of their subunits. In particular, Acxβ consists of ca. 720 amino acids, which is about 50 more than in Apcβ and about 150 more than in Hydβ orthologues. Most of the additional amino acids are located at the N- and C-terminal ends of the subunits. Acxα orthologues are ca. 30 amino acids longer than those of Apcα and more than 100 amino acids longer than Hydα orthologues. Finally, Acxγ orthologues contain ca. 50 more amino acids in their N-terminal regions, compared to Apcγ.

Fourth, a comparative structure-based sequence analysis revealed several signature motifs for the hydantoinase/keton carboxylase family at the interface regions of subunits, at the metal binding site of subunit γ containing four conserved cysteines, at the ATP binding sites of subunits α (and α′) and at the glycine-rich segments characteristic for the new protein fold in subunits β. However, a specific identification of Apc, Acx and Hyd based on structural differences i.e. of the substrate binding sites reflected in a consensus sequence failed. In particular, members of the Apc and Hyd branches are phylogenetically highly diverse. In addition, only few enzymes are biochemically characterized yet (in the case of the Apc branch only one) and an assignment of the function of more distantly related species has to be regarded with caution.

After annotating specific family members as Apc, Acx or Hyd based on items 1–3, preliminary structural models for the AcxABC and HydAB complexes can be calculated on the basis of the established structure of the Apc core complex and sequence identities between the subunits of 20–30% i.e. with the Swissprot server^37^ (sFig. 3). In analogy to Apc, the structures are predicted to contain two central Acxβ or Hydβ subunits which form the dimerisation interfaces between the protomers Acxαβγ and Hydαβ. The subunit equivalent to Acxα or Hydα in Apc is Apcα and definitively not Apcα′ because Apcα′ does not form any contact area to Apcβ. In addition, the interface regions between Apcα and Apcβ are conserved in the Acx and Hyd sequences, whereas the corresponding sequence segments in Apcα′ deviate substantially. A fingerprint motif distinguishing Apcα and Apcα′ is found in segment R/KERiDsxG of the α/β domain of Apcα located adjacent to the interface with Apcβ which is not conserved in Apcα′. In line with the X-ray structure of Apc, subunit γ is apparently not essential for the integrity of the core complexes. Because of its absence in any Hyd complex this subunit is also not required for the ATP-dependent phosphorylation of amidic carbonyl groups by Hydαβ. The large distances between the subunits γ and the putative active sites of Acx or Apc make their direct participation in the carboxylation reaction unlikely either.

While the structure of the Apc core complex provides plausible insights into the functionality of the kinase-related partial reactions of the enzymes of the hydantoinase/ketone carboxylase family, the partial reactions related to carboxylation in Acx and Apc substantially differ as suggested by structural and biochemical data^10^. The structure-inspired mechanistic proposal for acetophenone carboxylation by Apc with Apcα′ and Apcε as central components cannot be directly applied to Acx due to the absence of subunit α′ from the core complex and the lack of the extra subunit subunit ε. Therefore, Acx cannot bind and activate the ketone and bicarbonate simultaneously in separate activation sites as Apc and thus the reaction must proceed sequentially. This prediction is consistent with the observed stepwise hydrolysis of ATP to ADP and further to AMP linked to the activation of acetone followed by that of bicarbonate in members of the *Xanthobacter* Acx subbranch^12^,^13^,^14^,^15^ (Fig. 1b). In contrast to Apc, both characterized biochemical variants of Acx exhibit uncoupled ATP hydrolysis only with the ketone, but not with bicarbonate as single substrate^12^ which can be correlated with a sequential reaction mechanism on a single ATP-binding subunit. The mechanistic differences of Acx vs. Apc may also correlate with the higher expected stability of the enolphosphate derivatives of aliphatic ketones compared to those of aromatic ketones, because the latter cannot easily isomerise to a conformation with a double bond extending towards the aromatic ring. Further studies are necessary to work out the intricate catalytic cycle for carboxylating these inert ketones and to unravel the astonishing mechanistic differences among ketone carboxylase reactions despite highly related architectural features and reaction types.

## Methods

### Cultivation and purification

Cultivation and harvesting of ethylbenzene-degrading *A. aromaticum* cells and purification of the Apc core complex were performed as described previously^10^. Activity of the Apc holoenzyme (purified native Apc core complex substituted with recombinantly overproduced Apcε) was confirmed by determination of the ATPase activity and carboxylation of acetophenone with [^14^C]-bicarbonate to [^14^C]-benzoylacetate^10^. The Apc core complex was concentrated to a solution containing 40 mg ml^−1^ enzyme in 10 mM Tris buffer, pH 7.5, containing 0.1 M KCl and 5 mM acetophenone and used for crystallization.

### Crystallization and structure determination

**Figure 4:**
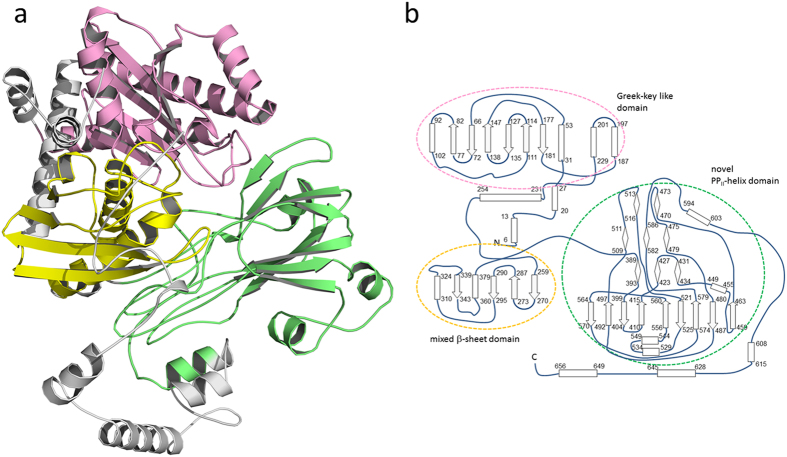
The Apcβ fold. (**a**) Structure of Apcβ composed of three domains. The N-terminal Greek-key like domain (pink) and the mixed β-sheet domain (yellow) are architecturally most related to the gliding protein MglB (pdb-code: 3T1R) and an ornithine acetyl transferase domain (pdb-code: 1VZ6), respectively. A domain with a novel fold (green) can be subdivided into a β-barrel and a polyproline II-like helix bundle. Apcα is characterized by long N- and C-terminal arms and extended interdomain linkers (in grey) mostly consisting of helices. (**b**) Topology diagram of Apcβ. The domains are bordered by circles with colors as in a.

**Figure 5:**
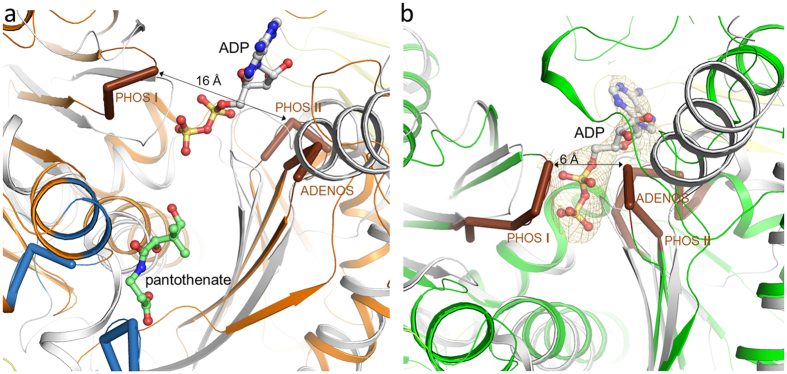
The ATP-binding sites. The conformations of the known signature motifs for ATP binding in ASKHA domains, ADENOSINE (GxxPGP), PHOSPHATE 1 (DxGGTxDDT) and PHOSPHATE 2 (DVGGT) highlighted in brown, are strictly conserved in Apcα and Apcα′, as well as in Acxα and Hydα. (**a**) Superimposed structures of the ATP and substrate binding sites of pantothenate kinase (gray) and Apcα (orange). Apcα is in an open state. The architecture of the ATP binding site is well conserved, although, in addition, loop segments α406–α417 and α691–α692 from a two-helix protrusion of the ASHKA module and from the C-terminal domain of Apcα, respectively, contribute to form the ATP binding site in Apcα. The predicted substrate binding site of Apcα is formed by the loop α279–α281 at the N-terminus of helix α282:α289, segment α333–α334 and Leuα345 of the central β-strand and helix α79:α87 (highlighted in blue). (**b**) Superimposed structures of the ATP and substrate binding sites of pantothenate kinase and Apcα′. The ATP binding site of Apcα′ (green) is in a closed state. The predicted substrate binding site is primarily formed segment α′70–α′77, the N-terminal loop of helix α′251:α′265 and the segment α′275–α′282. ADP is shown in a ball-and-stick representation (carbon in gray). The 2F_obs_ − F_calc_ electron density (brown) of ADP is drawn at a contour level of 0.9 σ.

**Figure 6:**
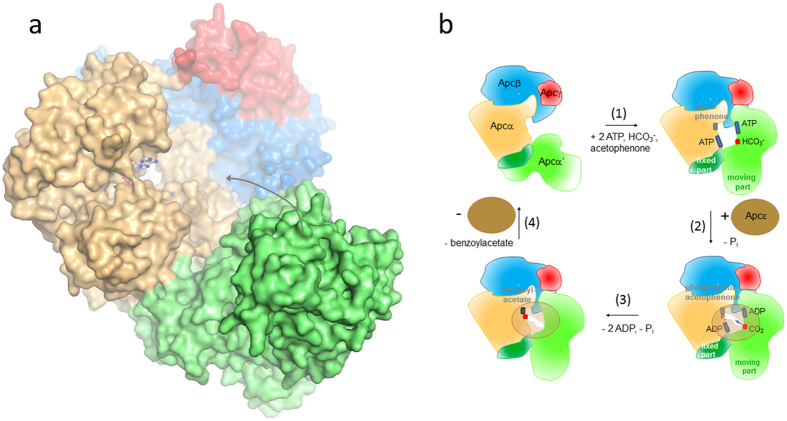
Scheme of the proposed enzymatic mechanism of Apc. (**a**) Molecular surface representation of one Apcαα′βγ protomer in the structurally obtained open form. We postulate that upon substrate binding Apcα′ (green) moves towards the wide hollow formed by Apcα (beige), Apcβ (blue) and Apcγ (red). (**b**) Reaction cycle (1) Binding of ATP (blue) and acetophenone (grey) to Apcα (beige) as well as ATP (blue) and HCO_3_^−^ (red) to Apcα′ induces a large conformational change of the apparently highly flexible part of Apcα′. (2) Binding of Apcε (brown) triggers the two kinase reactions. (3) Carboxyphosphate or the CO_2_ produced after hydrolysis diffuses via a formed channel (arrow) to the phosphoenol acetophenone, and the carboxylation reaction proceeds. (4) Dissociation of the Apc complex, product release and transition of the Apc core complex from closed to open.

Crystallization of the Apc core complex was performed by the sitting drop method at 4 °C with diverse screening solutions (JBScreen Classic 1–10, Pentaerythritol Screen, JCSG Core Suite I–IV, Morpheus Screen) using the Cartesian Honey Bee robot system. The optimized conditions are listed in Table 1. Freshly grown crystals normally diffracted to ca. 4 Å resolution but after long-term storage (up to 2 years), the resolution increased to ca 3.5 Å, possibly due to dehydration effects. After soaking with 0.2 mM mersalylic acid a single crystal diffracted to ca. 3 Å resolution. Anomalous data at the HgL_III_ edge with the peak wavelength of 1.008 Å were collected at the beamline PXII of the Swiss-Light-Source in Villigen (Switzerland) and processed with the program XDS^38^ (Table 1). The overall data quality was only satisfying to a resolution of 3.3 Å. However, spots were clearly visible at 3.0 Å resolution, although blurred in the quality parameters (Table 1) due to radiation damage and weak anisotropic diffraction properties. The space group is *P*6_5_22 and the cell axes are 240.1 Å and 336.6 Å, best compatible with an Apc(αα′βγ)_2_ heterooctamer in the asymmetric unit. The positions of three anomalously scattering Hg atoms in the asymmetric unit were detected with the programs SHELXC/D^39^. Initial phase determination was performed with SHARP^40^ at 4.0 Å resolution by the SAD method and subsequent solvent flattening with SOLOMON^41^ at 2.8 Å resolution. Inspection of the resulting electron density only revealed one Apcαα′βγ protomer in the asymmetric unit, which corresponds to a solvent content of 79%. Apcα, Apcβ and Apcγ could be partly traced automatically by PHENIX^41^ and BUCCANEER^42^,^43^, while Apcα′ was entirely traced manually using COOT^44^. The extraordinary high B-factor of 137 Å^2^ of Apcα′ substantially complicated the fitting of the polypeptide into the solvent-flattened electron density map, which was only feasibly because of its structural relationship to Apcα. The particularly low quality of the electron density for Apcα′ reflects its high mobility compared to Apcαβγ which might even be higher in solution because of the absence of an observed crystal lattice contact to Apcα′of a neighboring complex.The resulting model is reminiscent to that of many membrane proteins in exhibiting a high temperature factor of the coordinates (B_aver_ = 109 Å^2^). Refinement was performed with REFMAC^45^ and PHENIX^42^. Rigid body thermal displacement was taken into account by five TLS (translation, libration, screw) groups. The final R/R_free_ factor was 20.2/24.0% in the resolution range 50.0–2.84 Å (Table 1). The geometric quality of the model was assessed with MOLPROBITY^46^ and COOT. Figures 2, 3a, 4a, 5 and 6a were produced with PYMOL (The PyMOL Molecular Graphic System, Schrödinger, LLC.).

### Sequence analysis

Sequence similarities were analyzed by using BLAST (NCBI, Bethesda, MD), and phylogenetic trees were constructed using Clustal omega (EMBL-EBI, Cambridge, UK) and iToL (EMBL, Heidelberg, Germany).

### Data availability

Atomic coordinates and structure factors for the crystal structures are deposited in the protein databank under the accession code 5L9W.

## Additional Information

**How to cite this article**: Weidenweber, S. *et al*. Structure of the acetophenone carboxylase core complex: prototype of a new class of ATP-dependent carboxylases/hydrolases. *Sci. Rep.* **7**, 39674; doi: 10.1038/srep39674 (2017).

**Publisher's note:** Springer Nature remains neutral with regard to jurisdictional claims in published maps and institutional affiliations.

## Supplementary Material

Supplementary Dataset 1

## Figures and Tables

**Table 1:** Crystallographic data of the Apc core complex from *A. aromaticum*.

Crystal	Apc_highres	Apc_peak
Crystallization conditions	34–50% (w,v) PEE 797, 0.1 M Hepes pH 7.5	
Soaking conditions	5.5 h, incremental addition of mersalyl acid to a final concentration of 0.76 mM	
Data collection		
Space group	*P*6_5_22	*P*6_5_22
Wavelength [Å]	1.008	1.008
Resolution range [Å]	50.0–2.8 (2.9–2.8)	50.0–3.3 (3.4–3.3)
Unit cell *a*; *b; c* [Å]	240.1, 336.6	240.1, 336.6
Redundancy	13.7 (11.2)	14.0 (13.4)
Completeness [%]	95.8 (90.2)	99.9 (99.9)
R_sym_ [%]	11.3 (711.9)	12.9 (179.2)
I/σ(I)	25.0 (0.5)	22.4 (2.2)
CC (1/2)	100.0 (59.2)	99.0 (79.8)
Refinement statistics		
Apc(αα′βγ)_2_ in asym. unit	1/2	
No. residues,	α: 8–718; α′: 1–654;β: 2–684; γ: 1–127	
ADP, Hg, solvent,	1, 4, 15	
R_working_, R_free_ (%)	20.2, 24.0	
B_average_ (Å^2^)		
polypeptide, ADP	109, 170	
Disordered segments	α′526–α′530	
R.m.s. deviation		
bond lengths (Å)	0.007	
bond angles (°)	1.01	
Ramachandran Plot		
favored, outliers (%)	93.1, 0.3	
