# Supplementary material for: Structure of the acetophenone carboxylase core complex: prototype of a new class of ATP-dependent carboxylases/hydrolases
Source: Sci Rep. 2017 Jan 5;7:39674. doi: 10.1038/srep39674 (PMC5214803; doi:10.1038/srep39674)
Supplement: Supplementary Dataset 1 [file srep39674-s1.doc]

**Structure of the acetophenone carboxylase core complex: prototype of a new class of ATP-dependent carboxylases/hydrolases**

Sina Weidenweber^1^, Karola Schühle^2^, Ulrike Demmer^1^, Eberhard Warkentin^1^, Ulrich Ermler^1*^ & Johann Heider ^2*^

*^1^Max-Planck-Institut für Biophysik, Max-von-Laue-Str. 3, 60438 Frankfurt am Main, Germany.*

*^2^Laboratorium für Mikrobiologie, Fachbereich Biologie and SYNMIKRO, Philipps-Universität, 35032 Marburg, Germany.*

* Correspondence and requests for materials should be addressed to J.H. (email: [heider@staff.uni-marburg.de](mailto:heider@staff.uni-marburg.de)) and U.E. (email: ulrich.Ermler@biophys.mpg.de)


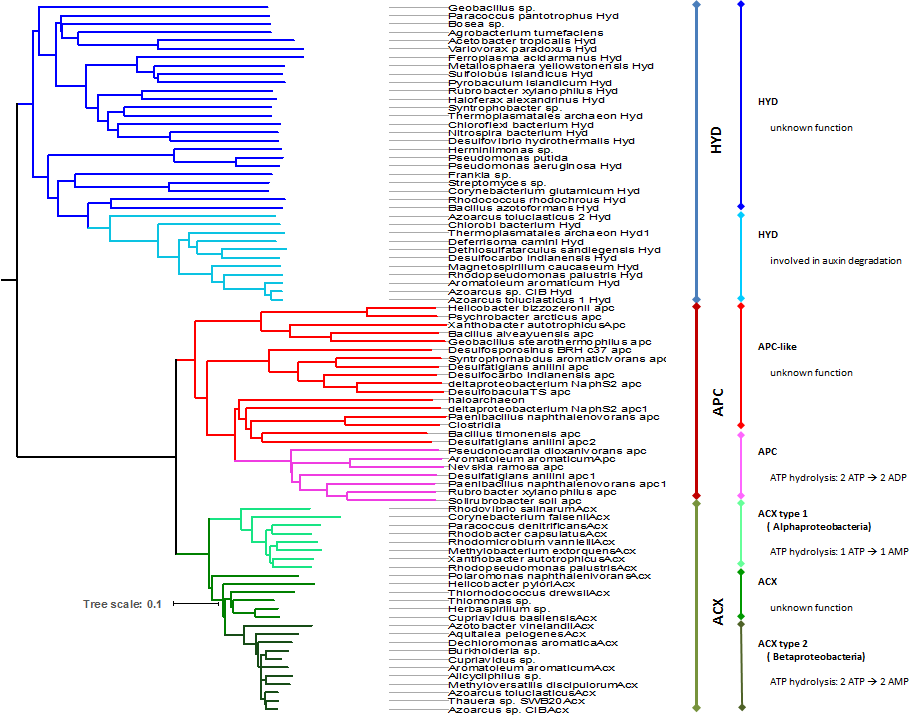


**Supplementary Figure 1 | Phylogenetic tree of the hydantoinase/ketone carboxylase enzyme family.** Sequence alignment is based on 85 sequences of β subunits. 24 Apc or Apc-like (in red), 25 Acx or Acx-like (in green) and 36 Hyd or Hyd-like (in blue). Accesion numbers: Apc or Apc-like (WP_011237167, WP_022977954, WP_062408325, WP_062408321, WP_028320072, WP_051184386, WP_028321589, WP_013677935, WP_011564818, WP_006422096, WP_028066685, WP_010283666, ADJ94044, WP_021072817, KUO77149, WP_028895878, WP_031451030, WP_006420123, WP_049674893, WP_044894653, CCB79032, WP_011280716, WP_043905618, gb|ABS66590); Acx or Acx-like (WP_011238463, ABS68739, WP_050417507, WP_018987852, WP_043743187, WP_039012057, WP_019918103, WP_059400977, WP_011286784, WP_028370428, WP_062788552, WP_031404665, WP_040039855, WP_041807136, WP_011800353, WP_007040689, WP_001285110, WP_017230923, WP_012606159, WP_011474023, WP_013421015, WP_011750491, WP_023916329, WP_052337556, WP_027288309); Hyd or Hyd-like (WP_011236982, WP_011565352, WP_049676698, WP_050415547, WP_018991579, WP_018991597, WP_011664628, WP_008618704, WP_044350772, EMR74873, WP_025322207, KXK04905, WP_051446073, WP_035195012, WP_051851708, WP_061285285, EFC82761, WP_023048099, WP_025916873, WP_031690354, WP_035377388, WP_021004500, KAJ32574, KPJ69355, WP_043232212, WP_006600969, KYK34611, WP_036747237, KXK22995, WP_012749113, WP_012715992, WP_048087598, WP_011761891, WP_009887040, KPK30924, WP_027177353).


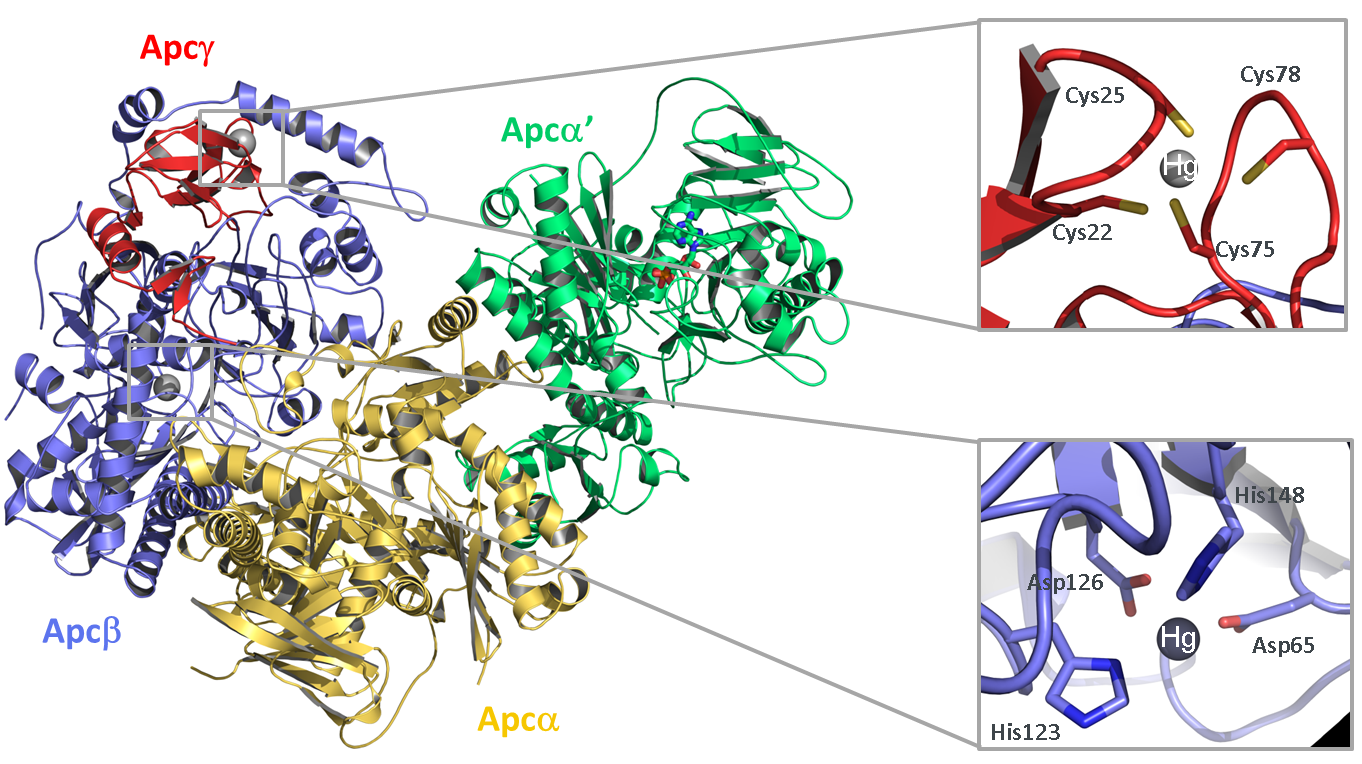


**Supplementary Figure 2 | Potential Zn^2+^ binding sites.** The Apcαα’βγ protomer shows two potential Zn^2+^ binding sites which are occupied by Hg(II) in the structure obtained after soaking with mersalylic acid. We suggested the first Hg^2+^ as the more plausible Zn^2+^ binding site. The second Hg^2+^ binding site in Apcβ may also be physiologically relevant, if significant amounts of Zn^2+^ or another metal ion were lost during purification, or may be related to the observed inhibition of Apc activity by low concentrations of additionally added Zn^2+^ ions.


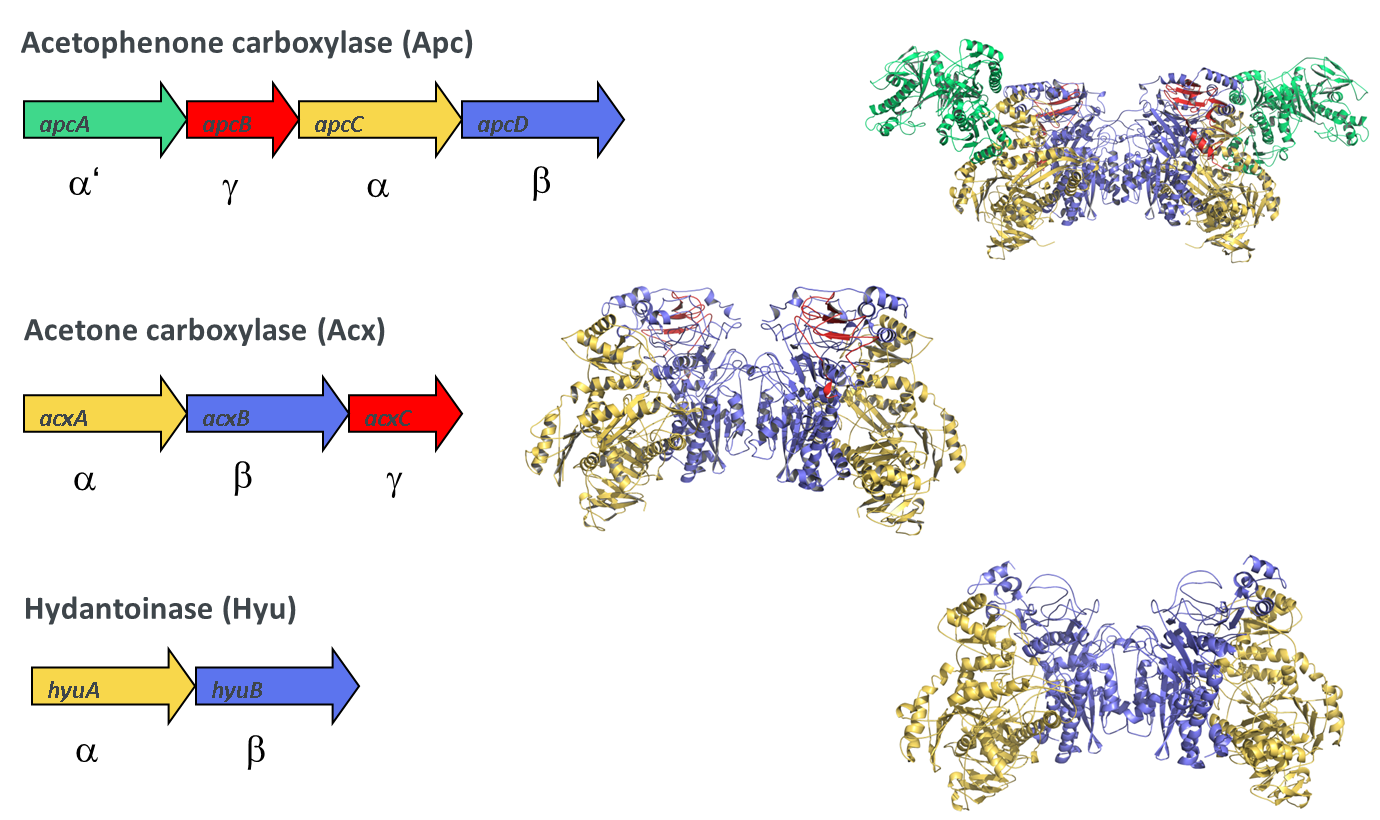


**Supplementary Figure 3 | Models of Acx and Hyd structures derived from the Apc structure by the Swiss-Prot server.** Gene arrangement in the genomes of Apc, Acx and Hyd. For modelling we used the Acx sequence of *Rhodobacter capsulatus* and the Hyd sequence of *Pseudomonas aeruginosa*.
